# Valencia’s Cathedral Church Bell Acoustics Impact on the Hearing Abilities of Bell Ringers

**DOI:** 10.3390/ijerph16091564

**Published:** 2019-05-04

**Authors:** Laura García, Lorena Parra, Blanca Pastor Gomis, Laura Cavallé, Vanesa Pérez Guillén, Herminio Pérez Garrigues, Jaime Lloret

**Affiliations:** 1 Instituto de Investigación para la Gestión Integrada de zonas Costeras, Universitat Politècnica de València, C/Paranimf 1, Grau de Gandia, 46730 Valencia, Spain; laugarg2@teleco.upv.es (L.G.); loparbo@doctor.upv.es (L.P.); 2 Sección de Otoneurología del Hospital Universitario La Fe, 46026 Valencia, Spain; bpastorgomis@gmail.com (B.P.G); cavallelaura@gmail.com (L.C.); perez_mva@gva.es (V.P.G.); perez_her@gva.es (H.P.G.)

**Keywords:** audiometry, acoustics, bell ringers, church bells, Valencia cathedral

## Abstract

Studies on the effect of occupational noise have been widely performed for occupations such as construction workers, workers of factories or even musicians and workers of nightclubs. However, studies on the acoustics of church bells are very scarce and usually reported in languages other than English. In Spain, although the tradition of bell ringers is progressively getting lost, some bell ringers that continue transmitting the tradition remain. Church bells create sound with a large sound pressure level that can be heard from a great distance. However, despite the characteristics of the sound of church bells, bell ringers do not present symptoms of occupational hearing loss unlike musicians and construction workers. To determine the effects of the sound of the church bells on bell ringers, in this paper, an acoustic study of the church bells and a physiological study of the hearing abilities of bell ringers. Results show sound pressure levels reaching 120 dB inside the bell tower. The resulting hearing loss in bell ringers is small considering the great intensity of the sound produced by the bells. This is likely due to the short amount of time that bell ringers are exposed to the sound even if it reaches high sound pressure levels.

## 1. Introduction

The acoustic trauma or Noise-Induced Hearing Loss (NIHL), is defined as the permanent functional damage in the ear caused by the exposure to high intensity sounds. It can emerge in an acute manner after a single exposure to intensities higher than 140 dB, or chronically by a maintained exposure to more than 80 dB. The NIHL is a sensorineural hearing loss (SNHL) that initially manifests as a loss in 4000 Hz. As the NIHL progresses, the damage affects all high frequencies. In the tonal audiometry with extension in high frequencies, the repercussion is evidenced in frequencies higher than 8000 Hz, predominantly between 14,000 and 16,000 Hz. Usually, hypoacusis is bilateral and symmetric. However, if the exposure is acute, it is possible to find one sole affected ear. Furthermore, the association between tinnitus and otalgia after the exposure is frequent. 

The factors that contribute to the NIHL are dependent both on the sound and the subject. Intensities higher than 80 dB, extended exposure times, the frequency of the noise (being frequencies between 2000 and 3000 Hz the most harmful), the type of noise (where discontinuous or intermittent noises generate the most damage), and the environment (where a greater damage is caused when the exposure takes place in enclosed venues with strong reverberation or inadequately soundproofed venues) are some of the factors that depend on the noise. The factors that depend on the subject include sensitivity of the individual, age and otological background.

Most of the published papers on acoustic traumatism evaluate hearing loss produced by irregular and disharmonic sounds [1,2,3,4,5]. However, musical sounds, being harmonic and regular, may be especially intense and produce hypoacusis. Several publications conclude that there is acoustic trauma in professional musicians [6,7].

Construction of the bell tower of the Cathedral of Valencia, known as the Micalet or Miguelete finalized in 1415. Its height is 50.85 m (61 if the final embellishment is considered) and its longitude is the same as the perimeter of its base. The inside of the tower is accessed through a spiral staircase. There are 13 bells on the tower, eleven of them in the bell room and two on the belfry. The bells of the belfry (Micalet, from 1539 and Quarts, from 1736) are dedicated to marking the hours and are currently activated electronically. Therefore, they have been excluded from this study.

The architectural characteristics of the bell tower lead to the physical characteristics of the final sound generated by the bells being determinant in the results obtained from studying the hearing of the bell ringers. The tower, which has an octagonal base, has four bodies and a belfry. The walls are made of ashlar stone and have a width of 3.9 m. The bells are in the fourth body, housed in the openings to the outside that are on each side of the octagon. The measurements of said openings are 2 m of width and 7.6 m of height. The upper half of the opening is diaphanous, and the lower half is closed with wood doors that absorb the sound and project it in an amplified manner to the exterior and in a buffered manner to the interior. Similar to what happens to the acoustic box of a guitar. The ceiling of the bell room is pointed and its physique, and probably its height as well, influence the quality and the propagation of the sound. For the performance, the bell ringers are located on top of a wooden beam which requires good balance. Therefore, the bell ringers perceive a sound that results from the characteristics of each sound and the architecture of the Miguelete.

Throughout the year there are different performances corresponding to different religious events. There are performances where only one bell is rung and others where some of them or all of the bells are rung. Each bell ringer controls one bell. However, sometimes one bell ringer may control two bells. The bells are made of bronze. They were made between years 1440 and 1816 and weigh between 317 and 1980 kg. The intensity of the sound on the point where the bell ringer is situated is between 100 and 125 dB, depending on the bell. At the middle of the room, the intensity varies between 110 and 125 dB, depending on the number of bells that bell ringers are ringing. 

Bell ringers are repeatedly exposed to high levels of noise. However, these high sound pressures may not impact their auditive and/or vestibular system. Currently, the group of bell ringers of the Cathedral of Valencia is comprised of volunteers that enjoy the art of ringing bells and transmit their knowledge from generation to generation. Bell ringers do approximately 200 performances a year. Not all bell ringers perform at all the performances. The duration of each performance varies from 15 to 30 min. Some days there may be several performances with rest periods ranging from 30 min to several hours between each performance. For example, there may be a performance in the morning and another one in the evening. The oldest bell ringers accumulate 50 years of exposure and the youngest ones have been performing for 3 years. During the performance, bell ringers maintain a distance to the bell between 15 and 100 cm and, habitually, they do not use any hearing protection. The objective of this study is to evaluate the hearing and balance of the bell ringers of the Miguelete tower of the Cathedral of Valencia.

This paper is structured as follows: Section 2 presents the related work. The acoustic study is analyzed in Section 3. Section 4 depicts the physiological study. The discussion in presented in Section 5. Finally, the conclusions and future work are presented in Section 6.

## 2. Related Work

Physiological studies on the hearing ability of workers exposed to high levels of noise during their every-day activities at their workplace are quite abundant. Armando Carballo et al. presented in [1] a study on objective noise-induced hearing loss on construction workers who were usually exposed to noise due to their work. The study was performed with 150 construction workers and 150 people who were not exposed to noise on their workplace. The study considered factors such as symptoms related to their auditive ability, the type of hearing protection, or sociodemographic information. Results showed that workers with higher exposure to noise presented pathological audiograms. Furthermore, 94.1% of the workers that never wore protection presented audiometric abnormalities and workers who wore at least one type of protection presented more audiometric abnormalities than those who wore two types (earplugs and earmuffs). Lie et al. reviewed in [2] papers on noise-induced hearing loss in workers. 187 papers were considered for the article. Results showed that hearing loss caused by occupational noise is between 7 and 21%. However, the authors stated the difficulty of discerning between occupational hearing loss and age-related hearing loss. Intermittent sounds, as in bells, would let the inner ear have resting periods, even if the duration of these periods is very small. Whereas, with the continuous sound, the aggression is constant for the duration of the exposure time. The resting periods of time may allow the ear to recover or avoid getting hurt as much as with the continuous sound.

Lastly, preventive measures are leading to the decrease in occupational hearing loss in industrial countries. Stucken et al. presented in [3] a study on the epidemiology and pathophysiology of occupational-induced hearing loss. Authors commented on the effects of patient and environmental factors and the use of antioxidants as a form of protection. Furthermore, they stressed the importance of prevention programs to avoid permanent or temporary threshold sifts. Basner et al. presented in [4] a compilation of the findings in the effects of hearing loss and its impact on behavior during the period between 2011 and 2014. Both occupational and non-occupational noise is considered. Jobs in the wood industry and mining are the ones that presented higher risks of occupational hearing loss with a 27% and the construction industry with a 23.5% of hearing loss in workers. Studies have also been performed on musicians and workers on nightclubs due to their exposure to loud music. Furthermore, people attending sports events, fitness classes, concerts and going to pubs and nightclubs are as well at risk of hearing loss.

Another study on occupational hearing loss is performed by Kovalova et al. in [5]. The study was performed with 4988 participants. Results showed that exposure to occupational noise in females did not present a significant difference from females not exposed to noise. However, males did present a significant difference although mean levels remained below 10 dB. Therefore, authors express the importance of using hearing protective equipment as a preventive measure. Morais et al. performed in [6] a study on hearing loss on music players. The study was performed on 65 musicians of the Symphonic Orchestra of Castilla y León. Results showed more than double of the percentage of musicians with hearing loss for their age and the 4000 Hz frequency. Furthermore, musicians who play viola and violin present more hearing loss in the left ear. Furthermore, another study on hearing loss in 42 musicians of pop, rock and jazz by Halevi-Katz et al. in [7]. Results showed musicians with more experience presented tinnitus and higher thresholds at frequencies ranging from 3 kHz to 6 kHz. Hearing loss was more related to a higher number of weekly hours of practice than the number of years playing an instrument. Furthermore, utilizing hearing protections was not related to the amount of exposure to amplified music.

Although studying the effects of occupational noise on workers of the construction or music industry is rather common, the effects of the sound of church bells on bell ringers and the acoustic properties of church bells are a fairly unexplored, and more so in the English language. On 1984, Rossing performed a study on the acoustic of bells [8]. He stated that the first five harmonic partials of bells were tuned in the ratios 1:2:2 and 4:3:4, and the fifth partial was the note most people heard. It seemed to be common for bells as a characteristic, to present an antinode on the part where the clapper strikes. The metal of the inner surface of the church bells was removed by the tuner in order to acquire the desired sound. Therefore, the tuning and the size of the bell determine the frequencies of the sound of bells. The study of the sound characteristics of the “Maria Gloriosa” bell in St. Peter’s Cathedral in the German city of Bremen was performed by Vogt et al. in [9]. The authors presented some mathematical models to characterize the bell and compare the results of those models to the frequency ratios of the bell itself. Results show that the obtained ratios are very close to the real ones.

For this paper, the sound level of the group of bells of the Cathedral of Valencia was studied so as to assess the noise bell ringers were exposed to when performing. The sound of the bells was measured as a whole. Furthermore, physiological tests were performed to the bell ringers and a control group to determine the impact of their activities as bell ringers on their hearing abilities.

## 3. Acoustic Study

### 3.1. Data Acquisition

**Figure 1:**
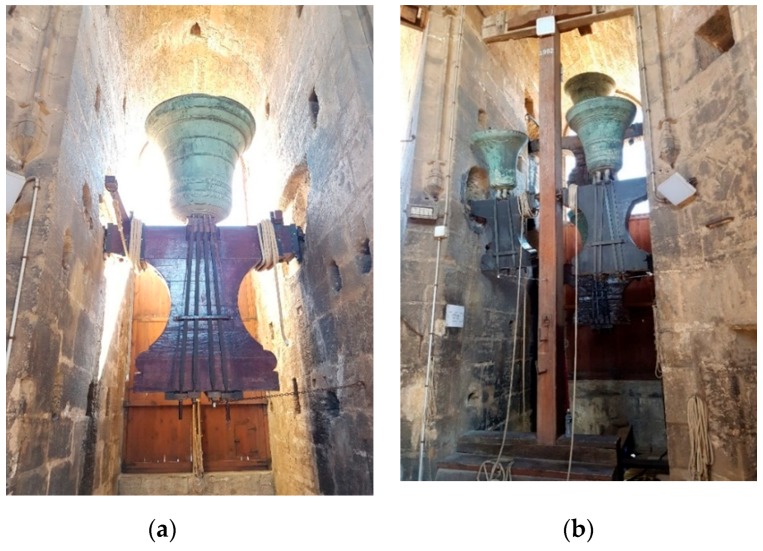
Images of the bells. (**a**) Single Bell. (**b**) Multiple bells located in one space.

**Figure 2:**
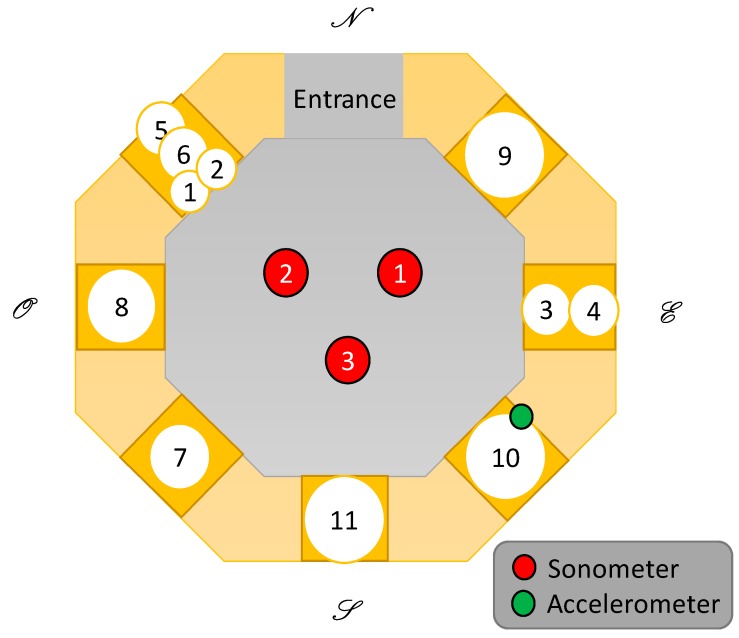
Deployment of the bells and position of the sonometers and the accelerometer.

In this subsection, the description of the bell tower and the deployment of the sonometers is presented. The bell tower of the cathedral of Valencia is a 50.85 m tall tower till its terrace and around 63 meter till its peak, made of stone with an orthogonal base. The bell room is comprised of eight windows where seven are occupied by bells and the last one is occupied by the stairs. The windows are provided with wood panels that act as an acoustic baffle. The information of the bells located at the bell’s room is presented in Table 1 [10]. The diameter of the bells ranges from 65 cm to 145 cm, and the weight ranges from 209 kg to 1980 kg. Some images of the bells are presented in Figure 1. Figure 1a is one of the larger bells while Figure 1b presents the smaller ones. The deployment of the bells is presented in Figure 2. All the areas in dark yellow represent the spaces where the bells and the windows are located. The areas in soft yellow are stone walls. The grey area is the central space of the room, where the bell ringers can move from bell to bell. Three sonometers were placed in the positions marked by the red dots in the direction of the bells located at the opposite side. An accelerometer was placed next to the bell “El Manuel” inside one of the square areas that can be seen on the walls adjacent to the bells.

**Figure 3:**
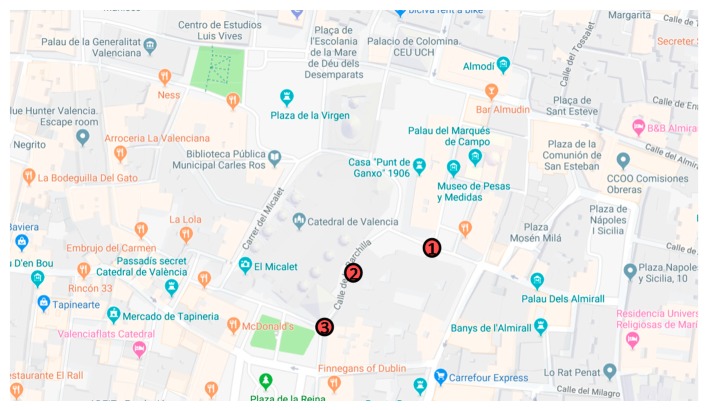
Position of the outside sonometers.

Measures were taken on the outside of the tower as well. Figure 3 presents the location of the outside sonometers. Other streets were blocked due to the festivities leading to the impossibility of placing the outside sonometers in other areas during the performance of the bells. The goal of these outside measures is to determine the sound level reached at different distances from the bell tower. The bell tower is represented in the map at the point with the name “El Micalet”.

### 3.2. Results

**Figure 4:**
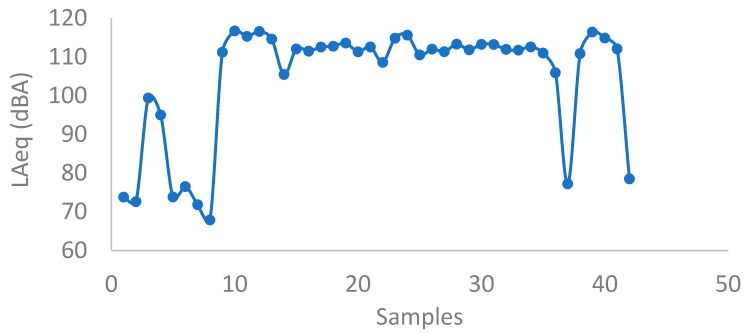
Evolution of the bell tower in dB(A).

In this subsection, the results from the inside and outside sonometers, as well as from the accelerometer, are depicted. Among the inside sonometers, one of them performed measures of a duration of 30 s, while the other two performed measures of 15 min. Figure 4 presents the equivalent continuous sound pressure level in dB(A), LAeq, of the sonometer that performed measures of 30 s. Therefore, measure 10 in the horizontal axis corresponds to 5 min after starting the measures. Measure 20 is corresponded to 10 min, measure 30 to 15 min and measure 40 to 20 min. As it can be seen, the equivalent sound pressure reaches levels above 110 dBA, with maximums of 116 dBA. It would be then obligatory, according to the Spanish law RD (Royal Decree) 286/06 for the workplace, which states that the lower, upper and limit equivalent sound pressures levels are 80 dBA, 85 dBA and 87 dBA, respectively, to use hearing protection and limit the exposure time.

**Figure 5:**
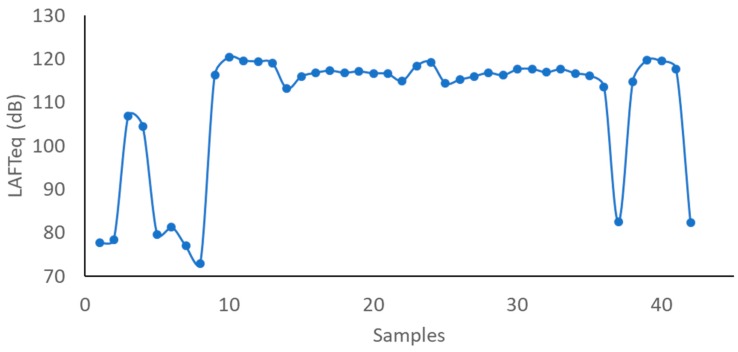
Evolution of the continuous sound pressure level in FAST mode (LAFTeq).

Figure 5 presents the equivalent continuous sound pressure level in dB(A) for the FAST mode. Results show that the LAFTeq can surpass 120 dBA in some moments.

**Figure 6:**
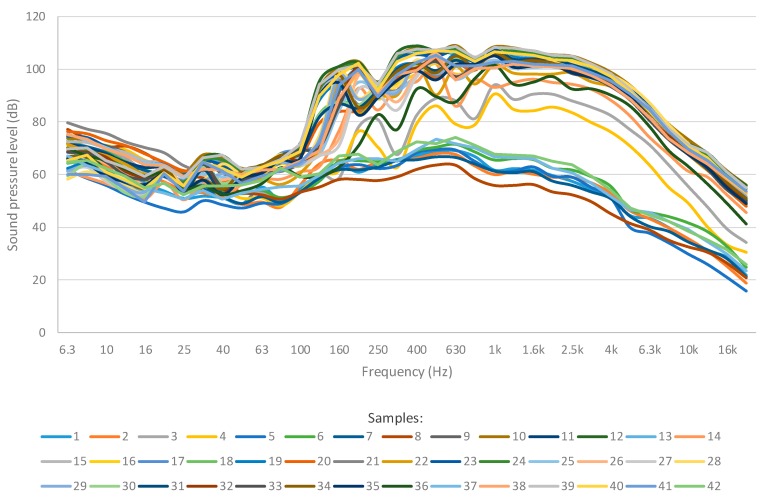
Evolution of the continuous sound pressure level for each frequency band.

The evolution of the continuous sound pressure level for each frequency band and each sample is presented in Figure 6. All bells rang during the performance for most of the time. However, there are some variations of the bells that were rung during the performance as seen in Figure 4 and Figure 5 that can be seen in the in the samples with lower sound pressures of Figure 6. As it can be seen, there are peaks that surpass 100 dB from 160 Hz to 4 kHz. Therefore, it is possible that bell ringers would be more affected at the frequencies from 160 Hz to 4 kHz.

For the other two sonometers, the continuous sound pressure level in dBA for a duration of the measure of 15 min is presented in Table 2. 

LAeq stands for the equivalent sound pressure levels measured in dBA. LAFmax is the maximum sound pressure level expressed in dBA and measured in FAST mode. The FAST mode is a time weighting characterized for a time of 125 milliseconds for both up and down. This time weighting is performed by the sound meter and is usually utilized to measure noise. Lastly, LAFTeq stands for maximum sound pressure level in one takt as defined in the standard DIN45641 and is provided by the sound meter as well. As it can be seen in Table 2, the maximum values surpass 120 dBA as for sonometer 1.

**Figure 7:**
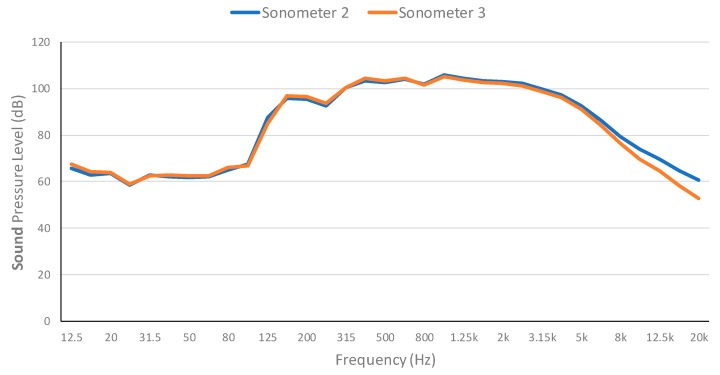
Evolution of the continuous sound pressure level for each frequency band (Sonometers 1 and 2).

Figure 7 presents the evolution of the continuous sound pressure level for each frequency band which is similar to that of sonometer 1. However, as the measures were taken for 15 min, the resulting graph presents lower equivalent sound pressure levels than those of the samples of Figure 6. Therefore, the equivalent sound pressure levels that surpass 100 dB comprise the frequencies from 315 Hz to 3.15 kHz.

**Figure 8:**
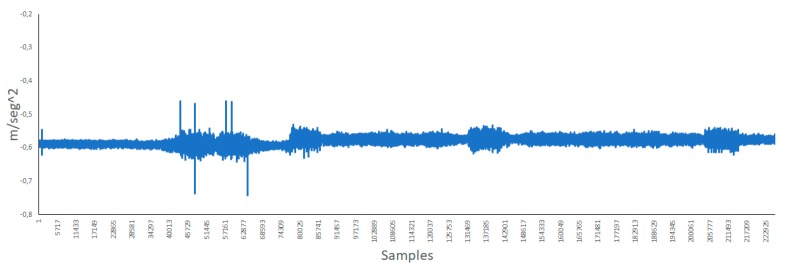
Data of the x axis of the accelerometer.

The vibration of walls and other materials may cause noise as well. Therefore, we performed some measures with an accelerometer so as to determine the vibration caused by the movement of the bells. Figure 8 presents the data of the x axis of the accelerometer. The beginning presents less fluctuations due to other bells being rung. However, when the bell closest to the position of the accelerometer starts being rung, the difference is appreciable. 

**Figure 9:**
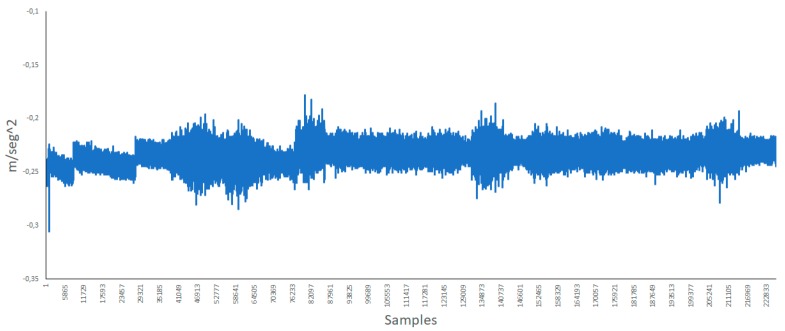
Y axis of the accelerometer.

Figure 9 shows the y axis of the accelerometer. In this axis, it can be seen the difference around measure 74,000 where all bells were ringing continuously. The vibrations in this axis is less pronounced than that in the other axis.

**Figure 10:**
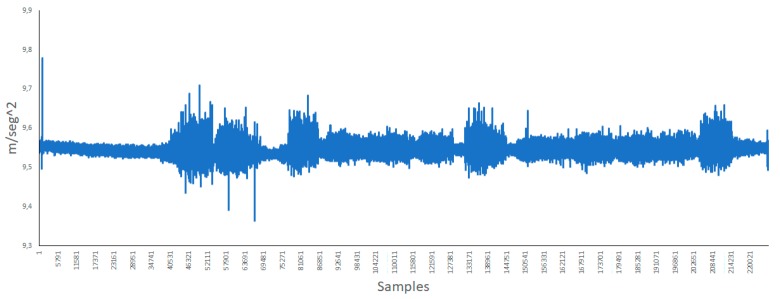
Z axis of the accelerometer.

Lastly, Figure 10 presents the z axis of the accelerometer. This is the axis that presents the most variations. 

The continuous sound pressure level in dBA for the three points where the outside measures were taken are presented in Table 3. These measures were taken so as to determine if the sound of the bells could be unpleasant to the people living in the surrounding areas. As it can be seen, the measures performed outside the bell tower did not exceed the pain threshold. Furthermore, due to the short duration of the performance and the time at which these performances occur, no damage can be caused to the habitants of the area. As opposed to the psychological damage caused by the bells of the churches that ring the hours at nighttime.

**Figure 11:**
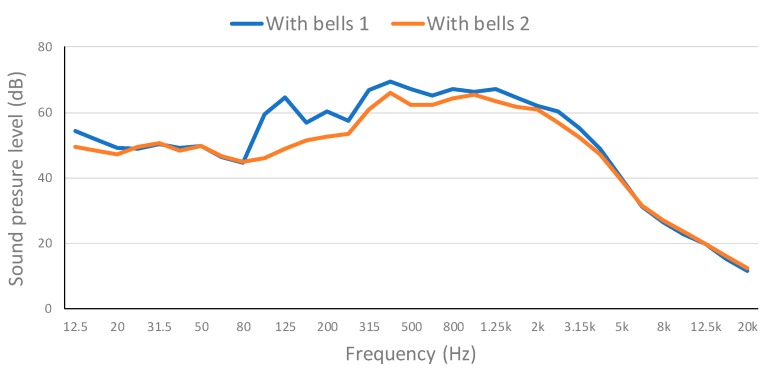
Evolution of the continuous sound pressure level for each frequency band in outside point 1.

The continuous sound pressure level for each frequency band at the Point 1 of the map is presented in Figure 11. Both measures were performed during the performance of the bells. Middle frequencies are the ones that present a greater sound pressure level. However, most of the time it remains under 70 dB and it never exceeds the pain threshold. Therefore, special bell ringing performances at the bell tower of the Cathedral of Valencia does not present any harm risk to the hearing of the neighbors. 

**Figure 12:**
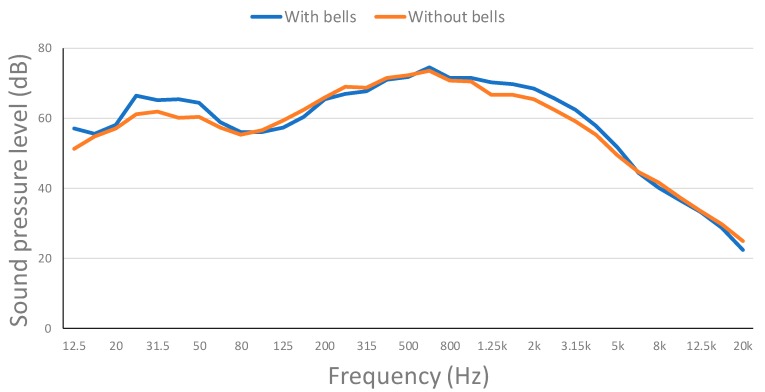
Evolution of the continuous sound pressure level for each frequency band in outside point 2.

The continuous sound pressure level for each frequency band at the Point 2 of the map is presented in Figure 12. Measures both with and without bells were taken. As it can be seen, measures without the bells are slightly below the measures with the bells. Middle frequencies are the ones with higher sound pressure levels, but they stay below the pain threshold.

**Figure 13:**
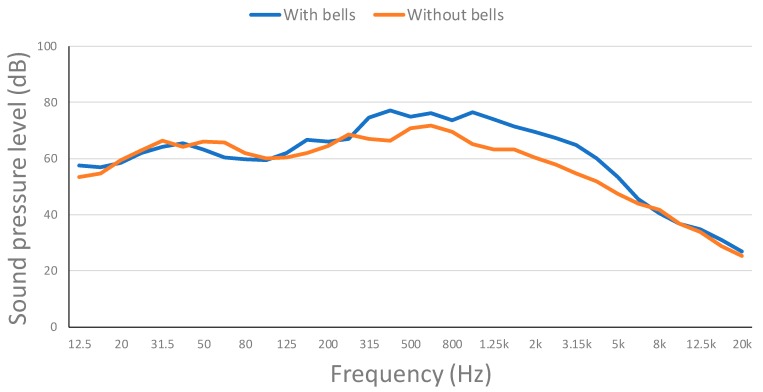
Evolution of the continuous sound pressure level for each frequency band in outside point 3.

The continuous sound pressure level for each frequency band at the Point 3 of the map is presented in Figure 13. Measures were performed with and without the bells as well. In this case, the difference is more evident, which can be related to the closeness to the source and the absence of obstacles. It is important to note that sound pressure levels continue to stay below the pain threshold.

## 4. Physiological Study

In this section, the physiological study on the hearing abilities of the bell ringers is depicted. The data acquisition process is explained as well as the characteristics of the subjects that participated on the study. Furthermore, the results are explained.

### 4.1. Data Acquisition 

In this subsection, the study performed on the bell ringers of the Cathedral of Valencia is going to be presented. The study has been performed in a tertiary hospital with the knowledge of the ethical committee. The study was performed with two groups being the experimental group and the control group. Table 4 presents the characteristics of the two groups. The participants were told that the aim of the research was to evaluate the effect of acoustic traumatism in the hearing and vestibular function. All subjects participated voluntarily and signed the informed consent. The experimental group was recruited from the group of bell ringers of the Cathedral of Valencia and is comprised of 17 men and 1 woman with a mean age of 43 ± 16 years old (standard deviation). The control group was comprised of healthy voluntaries among the medicine students and the hospital staff. A group without significant differences of age and sex in relation to the experimental group was created. The voluntaries were accepted regardless of the results of their hearing abilities. However, those who had a background of noise exposure or a hearing pathology were excluded.

The subjects included on the study were those that met the definition of an otologically normal subject: a person with normal health that does not present any sign or symptom of hearing disease, without cerumen plugs in the auditory canals and that has not been exposed in the past to excessive noises nor potential ototoxic medicines, nor presents a congenital hearing loss. The experimental group did not refer in any case to exposure to noise other than that of the bells.

Five ears, correspondent to three bell ringers, presented hypoacusis known before being a bell ringer. In two participants, the hypoacusis was neurosensorial bilateral and in one of them it was conductive unilateral. Those five ears were excluded from the study. None of the cases presented symptoms compatible with vestibular pathologies among their antecedents. However, the three patients with previous hypoacusis were excluded from the vestibular exploration.

### 4.2. Methodology

The methodology utilized to perform the physiological study is presented in this subsection. Specifically, a detailed anamnesis of the exposure to noise and ototoxics and, medico-surgical and occupational antecedents of the subjects was performed. Otomicroscopy was performed before the instrumental explorations. 

The audiological examination consisted in a tonal audiometry (in dB HL) with extension to high frequencies (up to 16,000 Hz), discomfort threshold, vocal audiometry, acoustic transitory oto-emissions and distortion products, tympanometry and stapedial reflex. The audiometry was performed with standard earphones. The stimuli were pure tones at all the frequencies using the recommended procedure Pure-tone air-conduction and bone-conduction threshold audiometry with and without masking, published by the British Society of Audiology [11]. It is important to use the same type of stimuli for both the control group and the experimental group in order to be able to compare the results. The thresholds in the conventional and the extended-high-frequency ranges showed good test-retest reproducibility [12]. Tympanograms were evaluated based on smoothness and symmetry. A normal tympanogram has a peak at approximately 0 daPa. The vocal audiometry has been performed using the records of bi-syllable words phonetically balanced from Cárdenas and Marrero. The object of the test of determination of uncomfortable loudness level (ULL) is to identify the minimum level of sound that is judged to be uncomfortably loud by the subject, according to the recommended procedure given in British Society of Audiology [13]. The used stimulus is a pure tone. The test is conducted on one ear at a time. Testing starts at 60 dB HL or at the subject’s hearing threshold level for that ear at that frequency, whichever is highest. A 1-second-long tone is presented followed by at least a 1-second quiet period. The stimulus is increased by 5 dB and presented in the same manner. The process is repeated. The level of the tone at which the subject responds is the ULL. 

The vestibular exploration consisted on a dynamic computerized posturography, cervical and ocular vestibular evoked myogenic potentials, video head impulse test and vibration-induced nystagmus with videonystagmograph recording. Table 5 lists the instruments that were utilized to assess the hearing abilities of the bell ringers.

Once the hearing thresholds were obtained, the correction of the hearing threshold was performed according to the Norma ISO 7029:2000 Distribución estadística de los umbrales de audición en función de la edad (Statistical distribution of hearing thresholds according to age) [14].

The calculation of the hearing threshold for an otologically normal population was performed for each age employing Equation (1):

∆Hmd, Y = α(Y − 18)(1)

where Y is the age and α is the coefficient of dB/year^2^ established for each age by the ISO normative. Afterwards, this value was subtracted from each hearing threshold obtained from the subjects. This way, the threshold corrected by age was obtained. Hypoacusis was established when the threshold corrected by age was above 20 dB.

The statistical analysis of the audiometric results was performed employing the SPSS 21 software program. A multivariate analysis of the variance (MANOVA) was utilized for frequencies between 125 and 8000 Hz. This way, we studied several dependent variables in the same analysis, avoiding the accumulation of type 1 error that occurs in each individual statistical analysis. The T of Student test for independent variables was utilized for each of the frequencies ranging from 9000 to 16,000 Hz.

### 4.3. Results

The results of the performed tests are presented in this subsection. Table 6 presents the hearing thresholds for the bell ringers. These thresholds seem to be unusually low because it is a relatively young sample population with only three people being more than 60 years old. Table 7 shows the values corrected by age. Table 8 shows the results of the control group without age correction. Lastly, Table 9 presents the results of the control group corrected by age.

**Figure 14:**
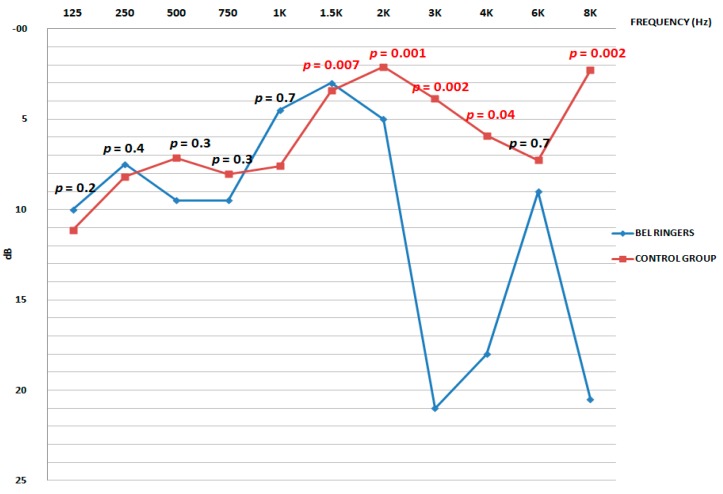
Average hearing threshold obtained from the audiometry of frequencies from 125 to 8000 Hz with age correction.

At the control group, the average auditive threshold corrected by age for conventional frequencies (125–8000 Hz) was below 10 dB for all frequencies. The auditive threshold for high frequencies was below 8 dB for all frequencies. The experimental group with the average auditive threshold corrected by age on conventional frequencies (125 Hz–8000 Hz) was below 20 dB for all frequencies, except for frequencies of 3000 Hz (21 ± 25 dB) and 8,000 Hz (21 ± 16 dB). The average threshold at 4000 Hz was 18 ± 13 dB (Figure 14). 

**Figure 15:**
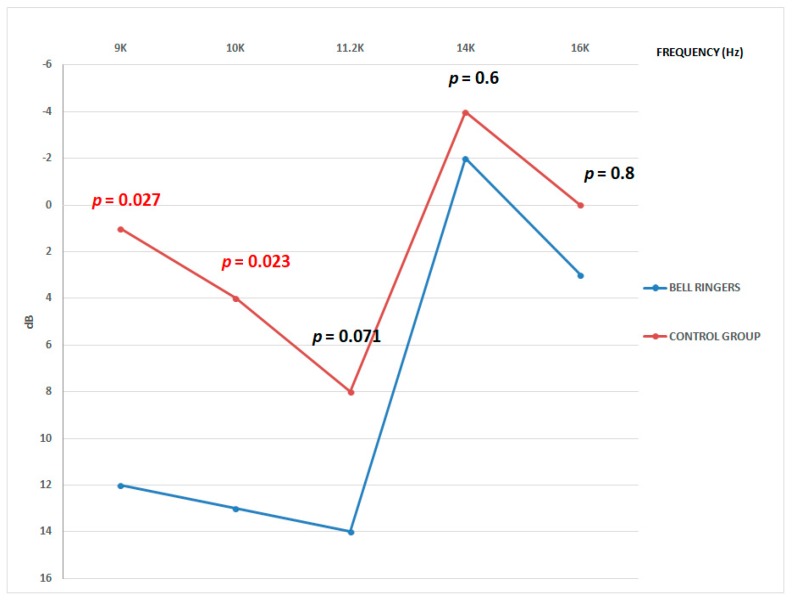
Average hearing threshold obtained from the audiometry with age correction for high frequencies.

The average auditive threshold at the auditive study employing a high frequency audiometer is presented in Figure 15.

The average discomfort threshold for frequencies of 250 Hz, 500 Hz, 1000 Hz, 2000 Hz and 4000 Hz was 105 ± 9 dB, 113 ± 12 dB, 112 ± 11 dB, 112 ± 11 dB and 111 ± 13 dB, respectively. The difference between the hearing threshold and the average discomfort threshold (dynamic range) for the aforementioned frequencies were 93 dB, 102 dB, 103 dB, 97 dB and 89 dB, being above 70 dB for all the subjects (Table 10).

The average threshold from where the participants were able to understand the 0%, 50% and 100% of the words from the vocal audiometry was 13 ± 4 dB, 18 ± 7 dB and 38 ± 14 dB, respectively.

According to the Jerger classification, a type A tympanogram (normal morphology with normal compliancy) for 24 ears (77.4%), type A_S_ (normal morphology with a decrease in compliancy) in 2 ears (6.5%) and a type A_D_ (normal morphology with an increase in compliancy) in 5 ears (16.1%).

The presence of stapedial reflex on all frequencies was confirmed for 26 ears (83.9%). The absence of stapedial reflex was detected at 2000 Hz for 1 ear (3.2%), at 4000 Hz for 2 ears (6.5%), at 5000 Hz for 1 ear (3.2%) and at all frequencies for 1 ear (3.2%) (Table 11). All the ears with negative stapedial reflex presented sensorineural hearing loss (SNHL) at the PTA (Pure tone audiometry) except one that presented negative stapedial reflex at 2000 Hz, being the PTA normal.

Transitory evoked otoacoustic emission (TEOAE) was registered in 28 of 31 ears (90.3%). No register was obtained from three ears (9.7%). Two of the ears with missing TEOAE had SNHL in the PTA: one ear with thresholds of 35–50 dB between 3000 and 6000 Hz and another ear with thresholds of 65 dB from 3000 Hz, affecting the entire range of high frequencies. One of the ears in which no TEOAE was recorded presented normoacusis in the PTA, being in this case type A_D_ tympanometry and the negative stapedial reflex in 2000 Hz, reflecting alterations in the middle ear that could be the cause of the absence of TEOAE.

Positive TEOAE were recorded in 18 ears with SNHL in the PTA: four ears with hearing loss from 16,000 Hz, two ears with hearing loss from 10,000 Hz and three ears with hearing loss from 8000 Hz, of which one ear presented thresholds of 35–40 dB in 3000 and 4000 Hz, three ears presented hearing loss from 4000 Hz and four ears presented hearing loss from 3000 Hz and affectation of the rest of the high frequencies. 

The majority of ears of the experimental group presented normal thresholds in the middle frequencies. Likewise, the presence of TEOAE demonstrates absence of lesion of the hair cells in this region of the cochlea. On the other hand, the presence of positive TEOAE did not rule out affectation of high frequencies from 3000–4000 Hz.

For the Distortion Product Otoacoustic Emission (DPOAE), six of 31 ears were negative (19.35%). DPOAE were recorded at all the studied frequencies in 25 of the 31 ears of the experimental group (80.65%). Positive DPOAE were obtained in 14 ears with SNHL in the PTA. Seven ears were affected by frequencies between 3000 Hz and 6000 Hz with thresholds between 20 and 50 dB and seven ears were affected by high frequencies from 8000–10000 Hz with normal thresholds in the rest of the frequencies. 

The DPOAEs were negative in the experimental group for six ears with SNHL in the PTA. Three ears had thresholds between 35 and 70 dB in 3000 Hz, 4000 Hz and 6000 Hz and affecting high frequencies, two ears with thresholds between 30 and 40 dB in the frequencies 1500 to 3000 Hz and rest of normal high frequencies and one ear with thresholds of 60 and 90 dB at 4000 Hz and 6000 Hz.

In the six ears in which DPOAE was not recorded, the hearing threshold in the PTA between the frequencies 2000 Hz and 6000 Hz was higher than the threshold of the ears with DPOAE. The mean threshold in the PTA in ears with positive DPOAE between 2000 Hz, 3000 Hz, 4000 Hz and 6000 Hz was 13 ± 6 dB, 19 ± 12 dB, 17 ± 11 dB and 20 ± 12 dB, respectively. The mean threshold in the PTA in ears with negative DPOAE between 2000 Hz, 3000 Hz, 4000 Hz and 6000 Hz was 28 ± 7 dB, 38 ± 12 dB, 43 ± 20 dB and 48 ± 32 dB, respectively.

In the experimental group, the absence of DPOAE correlated with hearing loss greater than 35 dB in the region of 3000 Hz to 6000 Hz, although in some ears with loss of 50 dB they were positive. Although the DPOAEs correlated better with the tonal thresholds between 2000 Hz and 5000 Hz than the TEOAE. Their presence did not rule out lesion in the hair cells of the basal regions of the cochlea (8000 Hz).

There are significative differences between the experimental group and the control group for 1500 Hz (*p* = 0.007), 2000 Hz (*p* = 0.001), 3000 Hz (*p* = 0.002), 4000 Hz (*p* = 0.049) and 8000 Hz (*p* = 0.020) frequencies (Figure 14). 

For these frequencies, the experimental group presents the highest auditive threshold. There have not been found any significant differences for the 125 Hz (*p* = 0.224), 250 Hz (*p* = 0.403), 500 Hz (*p* = 0.324), 1000 Hz (*p* = 0.770) and 6000 Hz (*p* = 0.068) frequencies. As it can be seen, there are points in the frequencies of 125 Hz and 1500 Hz were the hearing ability of the bell ringers is a little bit better. However, as it is shown in Figure 14, the difference is not statistically significant.

The size of the effect is a statistical instrument that shows the force of an exposition or intervention. This way, the inferential statistic is complemented, like the p value. In those frequencies where no significative differences have been found between the experimental group and the control group and we observe that the size of the effect is small. However, in those frequencies where a higher auditive threshold has been observed for the control group, we noticed that the size of the effect is very big, big or medium-big. Although no significative differences were found for the 6000 Hz frequency, we observe a medium size of the effect.

There are statistical significative differences for the 9000 Hz (*p* = 0.027), and 10000 Hz (*p* = 0.023) frequencies. For both cases, the effect has a medium-big size. Therefore, we can conclude that the exposure to the sound of the bells has an effect on the hearing for these frequencies. There have not been statistically significative differences for the 11200 Hz (*p* = 0.071) frequency. However, this value is near alpha and the size of the effect is medium. Lastly, there have not been found significative differences for the 14000 Hz and 10000 Hz frequencies, with a nearly non-existent effect size. (Figure 15).

For the vestibular exploration, the obtained gains in the video head impulse test (vHIT) were between 0.8 and 1.2 for the six channels in all subjects, and no out of correction events were observed.

Spontaneous nystagmus was not observed in the videoinstamography of any participant. Furthermore, nystagmus did not appear when applying vibration of incremental frequencies (30, 60 and 100 Hz) from the mastoids of any subject. Symmetric vestibular evoked myogenic potentials were observed in the 15 studied subjects.

The average value obtained at the sensorial organization test of the posturography was of a 82%, with a standard deviation of 8%. Two subjects presented a result below the normal limit for their age and size.

## 5. Discussion

In this section the obtained results of the physiological study are discussed. Acoustic trauma produces a SNHL that mainly affects high frequencies, with the scotoma at 4000 Hz, being the first finding in the tonal audiometry [15].

Multiple studies relate continuous exposure to noise in the workplace with hearing loss due to acoustic trauma [1,2,3,4,5], having found affectation in professional musicians [6,7]. After a thorough bibliographic search, we have not found any work related to audition in bell ringers. 

According to the published literature [16], the hearing loss at conventional frequencies (125 dB–8000 dB) in our study was lower than expected. Although there are statistically significant differences between the experimental group and the control group (at 2000–10,000 Hz), with the average hearing threshold being higher among the bell ringers, the loss is small. Likewise, greater affectation was observed in frequencies 3000 Hz and 8000 Hz than in the 4000 Hz frequency, characteristic of acoustic trauma. The reason of why bell ringers may not suffer much from hearing loss may be partly due to the sound pressure escaping through the big windows of the tower. Another part of it may be absorbed by the walls. Furthermore, the duration of the part of the performance when all bells are ringing at the same time does not exceed 15 min. It is also important to consider the different frequencies of the bells.

High frequencies intervene in the location of sound [17] and the understanding of language, especially in noisy environments [18]. They have also been related to Presbycusis, ototoxicity and acoustic trauma [16]. Different studies establish the usefulness of audiometry with extension in high frequencies in the early detection of hearing loss associated with ototoxics, mainly in chemotherapy trea TDH ents with cisplatin and, to a lesser extent, methotrexate and cyclophosphamide [16,19]. However, there is controversy about its usefulness in the detection of noise induced hearing loss, since we lack conclusive data at the present time. Some authors have found hearing loss due to acoustic trauma in the extension in high frequencies, predominantly at frequencies 14000 Hz and 16000 Hz [16]. In our study, we observed a higher frequency hearing loss than that found in conventional frequencies, with frequencies 9000–11200 Hz being the most affected. According to these results, high-frequency audiometry could be useful in the early diagnosis of acoustic trauma in patients in whom conventional frequencies have not yet been affected.

Otoacoustic emissions (OAE) are sounds of low intensity generated by the cochlea that leave to the middle ear and are detected in the external auditory canal. They are a product of the motility of the outer hair cells, which is why they represent a non-invasive objective technique for the study of preneural cochlear function, contributing to the differentiation between sensory and neural dysfunction [20]. The presence of OAE usually indicates normal hearing. However, OAE are affected by the external and middle ear pathology, so their absence does not necessarily indicate hearing loss. In general, OAEs will be absent when the cochlear hearing loss is greater than 30 dB [20]. OAEs can be spontaneous or provoked in response to an external stimulus. Of the latter, the most commonly used in clinical practice, and those evaluated in our study, are transient-evoked acoustic otoacoustic emissions (TEOAE) and distortion products (DPOAE). 

The TEOAE use as a stimulus a non-linear sequence of broadband tones or clicks with a sound pressure level of 70–84 dB SPL (45–60 dB HL) and stimulate a wide range of frequencies, between 1500 and 4500 Hz [20]. They are present in virtually all subjects with normal hearing. In different series, its presence is established in 98% of patients with normal hearing, leaving a small percentage of cases in which TEOAE is not recorded without hearing impairment [15]. The absence of registration may be due to anatomical variations in the external and middle ear, a defective registration technique or the inability of a specific stimulus to cause the onset of the otoacoustic emission. Exposure to noise and ototoxics produces a progressive modification of the TEOAE [20].

The DPOAEs are produced simultaneously using two tones of different frequencies: f1 and f2 (f2/f1 = 1.24), it being possible to configure f2 in a range of 1000 to 6000 Hz. The presence of otoacoustic emissions indicates that the outer hair cells are functioning in the frequency region of f2 [20]. In our study, we evaluated the 2000 Hz, 3000 Hz, 4000 Hz and 5000 Hz frequencies, considering the presence of DPOAE obtaining 2/3 positive records. The results are presented globally as positive / negative DPOAE as well as in the form of PDgram with DPOAE and sound level.

Differences were found with the general population in the vestibular study performed on the bell ringers, finding the average values obtained within the limits of normality for each age. Therefore, we cannot conclude that exposure to noise produces affectation at vestibular level.

## 6. Conclusions

Although the bell ringer tradition is getting lost, in the Cathedral of Valencia there are still several voluntary bell ringers that perform on special occasions. However, the high sound pressure levels given by the bells produce low hypoacusis in the bell ringers. In this paper, we performed an acoustic study of the sound produced by the bells and a physiological study of the hearing abilities of the bell ringers. The sound of the bells reached 120.32 dBA inside the bell tower and a maximum of 91.59 dBA outside the tower. The physiological results confirmed that bell ringers did not present hearing loss related to their occupation.

As a future work, we plan on studying the hearing abilities of the bell ringer of other churches and cathedrals in Spain so as to confirm the hypothesis presented in this preliminary study. The results of these studies could aid in developing hearing protections specific for bell ringers as in [21] so as to avoid damage in case of longer exposures to the sound produced by church bells. It could also be utilized to develop hearing health monitoring systems similar to the health monitoring system in [22] so as to add more profiles to the ones that have been more studied as construction workers or musicians.

## Figures and Tables

**Table 1:** Description of the bells.

Name of the Bell	Number	Diameter (cm)	Weight (kg)
L’Úrsula	1	65	209
La Violant	2	79	317
La Caterina	3	84	554
La Bàrbera	4	84	526
El Pau	5	90	422
L’Arcís	6	94	481
El Vicent	7	113	835
L’Andreu	8	129	1243
El Jaume	9	134	1750
El Manuel	10	139	1980
La Maria	11	145	1765

**Table 2:** Measures from inside sonometers.

Sonometer	LAeq (dBA)	LAFTeq (dBA)	LAFmax (dBA)
2	113.34	117.24	121.54
3	112.86	116.77	120.32

**Table 3:** Measures from outside sonometers.

Point	LAeq (dBA)	LAFTeq (dBA)	LAFmax (dBA)
**1**	72.07	76.04	80.39
**2**	79.96	83.95	87.79
**3**	83.01	87.48	91.59

**Table 4:** Characteristics of the subjects.

Group	Men	Women	Mean Age	Min Age	Max Age	Observations
**Experimental**	17	1	43	17	66	Bell ringers
**Control**	10	1	40	21	64	Healthy subjects

**Table 5:** Instruments employed to perform the physiological study.

Test	Instruments
Audiometry	Affinity 2.0 audiometer. Otoaccess ^TM^. Interacustics. Headphones TDH 39
Tympanometry	Madsen Otoflex impedanceometer. Otosuite 4.00.01. Otometrics.
Transitory acoustic oto-emissions and distortion product	Madsen AccuSreen. Type 1077. GN Otometrics A/S.
Posturography	Smart equitest posturographer. Natus.
Vibration-induced nystagmus	VVIB 3F vibratory stimulator. Synapsis.
Videonystagmograph	VNG Ulmer Synapsis, with CCD 1/3 iris inch automatic infrared monocamera, 320,000 pixels.
Video Head Impulse Test	ICS Impulse^R^. Otosuite Vestibular. Otometrics.
Vestibular evoked myogenic potentials	AEP ver 7.0.0 Navigator Pro, Bio-LogicSystems. Natus.

VNG: Videonistagmography. AEP: Auditory evoked potential.

**Table 6:** Hearing thresholds of the bell ringers.

Bell Ringers													
Hearing Thresholds in Conventional PTA													
Ear	Sex	Age	125	250	500	750	1000	1500	2000	3000	4000	6000	8000
1	M	48	20	15	15	15	10	10	15	45	35	20	20
2	M	24	10	5	10	10	5	10	10	15	10	15	15
3	F	64	25	15	20	15	10	20	30	35	35	50	40
4	M	30	15	10	5	5	5	5	10	10	10	10	5
5	M	27	20	20	15	15	10	20	15	20	15	20	15
6	M	65	15	15	20	30	25	35	30	60	55	70	70
7	M	59	10	10	5	10	5	5	10	15	10	15	15
8	M	30	15	10	10	5	5	10	5	5	5	5	5
9	M	29	15	15	10	20	20	35	30	40	20	15	10
10	M	17	15	10	10	5	5	5	0	5	0	10	5
11	M	52	20	10	10	10	5	10	10	25	20	30	55
12	M	47	20	15	15	15	10	15	15	25	15	15	15
13	M	66	15	10	10	15	5	10	10	20	35	50	45
14	M	36	20	20	15	15	5	10	10	15	15	20	20
15	M	45	15	10	5	5	0	5	5	5	5	5	25
16	M	51	10	5	10	15	10	15	15	30	60	95	75
17	M	48	15	3	10	15	15	10	15	45	35	15	20
18	M	24	15	10	10	10	10	15	15	15	10	15	15
19	F	64	10	5	5	10	5	15	30	35	50	35	25
20	M	27	15	20	20	25	15	20	25	20	15	15	15
21	M	65	15	10	20	20	20	20	25	40	55	55	65
22	M	59	20	7	15	15	15	10	20	15	15	20	20
23	M	30	10	5	5	5	5	10	20	10	15	15	15
24	M	29	15	15	15	20	25	40	35	25	15	15	10
25	M	17	10	5	5	5	0	5	0	0	0	10	5
26	M	52	20	12	15	15	15	15	15	15	15	25	35
27	M	47	15	8	5	10	10	15	5	40	35	20	15
28	M	66	15	10	15	15	10	15	15	20	35	45	40
29	M	36	20	20	20	15	10	15	15	15	15	25	25
30	M	45	10	3	5	5	5	5	5	10	10	15	20
31	M	51	5	2	10	10	5	5	5	15	25	25	55
AHT			13	9	13	13	8	8	10	30	30	23	38
SD			4	5	5	6	6	9	9	14	16	20	20

PTA = Pure tone audiometry. AHT = Average hearing threshold. SD = Standard deviation.

**Table 7:** Hearing thresholds of the bell ringers with values corrected by age.

Control Group													
Hearing Thresholds in Conventional PTA													
Ear	Sex	Age	125	250	500	750	1000	1500	2000	3000	4000	6000	8000
1	M	21	5	5	0	5	5	5	5	10	10	25	15
2	M	21	10	0	0	5	5	0	0	10	10	20	30
3	M	25	10	10	0	5	5	0	5	5	10	10	5
4	M	25	15	5	0	5	0	0	0	10	5	5	-5
5	M	25	10	5	5	10	15	10	5	0	10	5	0
6	M	25	10	5	0	0	0	10	20	0	−10	5	5
7	M	28	25	25	30	30	30	20	10	15	20	25	25
8	M	28	15	15	20	20	20	10	0	10	15	15	15
9	M	30	20	20	10	15	15	15	15	15	10	10	5
10	M	30	15	15	15	15	15	15	10	10	10	10	10
11	M	30	20	15	10	5	15	10	10	5	15	5	−5
12	M	30	15	10	5	10	5	0	0	0	0	5	0
13	M	46	10	10	25	20	15	10	5	15	25	30	15
14	M	46	5	5	5	10	10	0	0	15	25	25	55
15	M	50	5	5	10	10	10	0	0	5	5	10	5
16	M	50	5	0	10	5	5	0	−5	5	15	10	5
17	M	62	15	5	5	5	5	5	5	15	10	10	15
18	M	62	0	10	0	5	5	5	5	5	15	25	20
19	M	63	25	20	25	20	15	10	5	5	20	15	20
20	M	63	25	20	10	15	15	5	5	10	35	20	15
21	F	64	15	10	15	15	10	15	20	40	45	60	50
22	F	64	10	5	10	5	5	10	20	30	30	50	40
AHT		40	13	10	10	11	10	7	6	11	15	18	15
SD		17	7	7	9	7	7	6	7	9	12	14	16

PTA = Pure tone audiometry. AHT = Average hearing threshold. SD = Standard deviation.

**Table 8:** Hearing thresholds of the control group without correction by age.

Bell Ringers													
Hearing Thresholds in Conventional PTA Corrected by Age													
Ear	Sex	Age	125	250	500	750	1000	1500	2000	3000	4000	6000	8000
1	M	48	18	13	13	13	8	7	12	39	27	11	9
2	M	24	10	5	10	10	5	10	10	16	10	15	15
3	F	64	20	10	14	8	3	11	19	22	19	29	13
4	M	30	15	10	4	4	4	4	9	8	8	7	2
5	M	27	20	20	15	15	10	20	15	20	15	20	15
6	M	65	10	10	14	23	18	25	18	40	27	38	31
7	M	59	7	7	1	6	1	−1	3	3	−6	−3	−8
8	M	30	15	10	9	4	4	9	4	3	3	2	2
9	M	29	15	15	10	20	20	35	30	40	20	15	10
10	M	17	15	10	10	5	5	5	0	5	0	10	5
11	M	52	17	7	6	6	1	4	3	13	4	12	32
12	M	47	18	13	13	13	8	12	12	19	7	6	4
13	M	66	10	5	4	8	−2	0	−2	0	7	18	6
14	M	36	20	20	14	14	4	9	9	13	13	17	17
15	M	45	13	7	3	3	−2	2	2	−1	−3	−4	14
16	M	51	7	2	6	11	6	9	8	18	44	77	52
17	M	48	13	3	8	13	13	7	12	39	27	6	9
18	M	24	15	10	10	10	10	15	15	15	10	15	15
19	F	64	5	5	−1	3	−2	6	19	22	34	14	−2
20	M	27	15	20	20	5	15	20	25	20	15	15	15
21	M	65	10	10	14	13	13	10	13	20	27	23	26
22	M	59	17	7	11	11	11	4	13	3	−1	2	−3
23	M	30	10	5	4	4	4	9	19	8	13	12	12
24	M	29	15	15	15	20	25	40	35	25	15	15	10
25	M	17	10	5	5	5	0	5	0	0	0	10	5
26	M	52	17	12	11	11	11	9	8	3	−1	7	12
27	M	47	13	8	3	8	8	12	2	34	27	11	4
28	M	66	10	10	9	8	3	5	3	0	7	13	1
29	M	36	20	20	19	14	9	14	14	13	13	22	22
30	M	45	7	3	3	3	3	2	2	4	2	6	9
31	M	51	2	2	6	6	1	−1	−2	3	9	7	32
AHT			10	8	10	10	5	3	5	21	18	9	21
SD			11	8	5	5	5	6	10	25	13	3	16

PTA = Pure tone audiometry. AHT = Average hearing threshold. SD = Standard deviation.

**Table 9:** Hearing thresholds of the control group with values corrected by age.

Control Group													
Hearing Thresholds in Conventional PTA Corrected by Age													
Ear	Sex	Age	125	250	500	750	1000	1500	2000	3000	4000	6000	8000
1	M	21	5	5	0	5	5	5	5	10	10	25	15
2	M	21	10	0	0	5	5	0	0	10	10	20	30
3	M	25	10	10	0	5	5	0	5	5	10	10	5
4	M	25	15	5	0	5	0	0	0	10	5	5	−5
5	M	25	10	5	5	10	15	10	5	0	10	5	0
6	M	25	10	5	0	0	0	10	20	0	−10	5	5
7	M	28	25	25	30	30	30	20	10	15	20	25	25
8	M	28	15	15	20	20	20	10	0	10	15	15	15
9	M	30	20	20	9	14	14	14	14	13	8	7	2
10	M	30	15	15	14	14	14	14	9	8	8	7	7
11	M	30	20	15	9	4	14	9	9	3	13	2	−8
12	M	30	15	10	4	9	4	−1	−1	−2	−2	3	−3
13	M	46	8	8	23	18	13	7	2	9	17	21	4
14	M	46	3	3	2	8	8	−3	−3	9	17	16	44
15	M	50	2	2	6	6	6	−6	−7	−7	−11	−8	−18
16	M	50	2	−3	6	1	1	−6	−12	−7	−1	−8	−18
17	M	62	10	0	−1	−2	−2	−5	−7	−5	−18	−22	−24
18	M	62	−5	5	−6	−2	−2	−5	−7	−15	−13	−7	−19
19	M	63	20	15	19	13	8	0	−7	−15	−8	−17	−19
20	M	63	20	15	4	8	8	−5	−7	−10	7	−12	−24
21	F	64	10	5	9	8	3	6	9	27	29	39	23
22	F	64	5	0	4	−2	−2	1	9	17	14	29	13
AHT		40	11	8	7	8	8	3	2	4	6	7	2
SD		17	7	7	9	8	8	8	8	11	12	16	19

PTA = Pure tone audiometry. AHT = Average hearing threshold. SD = Standard deviation.

**Table 10:** Results obtained from the audiometry of frequencies from 125 to 8000 Hz with age correction.

Frequency	Average Discomfort Threshold	Difference between Hearing Threshold and Average Discomfort Threshold
250 Hz	105 ± 9 dB	93 dB
500 Hz	113 ± 12 dB	102 dB
1000 Hz	112 ± 11 dB	103 dB
2000 Hz	112 ± 11 dB	97 dB
4000 Hz	111 ± 13 dB	89 dB

**Table 11:** Results of the stapendial reflex.

**Presence of stapendial reflex**			
**Ears**		**Percentage**	
26		83.9%	
**Absence of stapendial reflex**			
**Frequency**	**Ears**		**Percentage**
2000 Hz	1		3.2%
4000 Hz	2		6.5%
5000 Hz	1		3.2%
All frequencies	1		3.2%
